# The Medical Treatment and Management of an Attack of Appendicitis—With a Review of Some of the Literature of the Subject Both Old and New*Read before the San Angelo (Texas) District Medical Society, February 10, 1903, and the Fourth District Medical Society of Texas, at Brownwood May 17, 1904.

**Published:** 1904-09

**Authors:** Boyd Cornick

**Affiliations:** San Angelo, Texas


For Texas Medical Journal.

### The Medical Treatment and Management of an Attack of Appendicitis—With a Review of Some of the Literature of the Subject Both Old and New.*

BY BOYD CORNICK, M. D., SAN ANGELO, TEXAS.

The word "appendicitis" is not found in my edition of Webster's Unabridged, which was issued from the press in 1880, before the word was coined. In "The American Dictionary and Cyclopedia," in sixteen volumes, published in 1900, it is correctly defined as "Inflammation of the vermiform appendix of the cœcum." And then follows a half-column of fine print descriptive of the affection, from which I quote the following extract: "The surgeons now regard the operation for its removal (meaning the removal of the vermiform appendix during an attack of appendicitis) as one of the most simple; but to obtain the best results it should take place a few hours after the patient begins to suffer from the disease. In

*Read before the San Angelo (Texas) District Medical Society, February 10, 1903, and the Fourth District Medical Society of Texas, at Brownwood May 17, 1904.

fact, the sooner the operation is performed the better are the chances of recovery, while *if neglected, death is sure to follow promptly;* or, after lingering, miseries from the deadly poison permeating the system and coming to the surface in abscesses."

The article from which the foregoing extract is taken is unsigned, but the title page of the American Dictionary and Cyclopedia contains a partial list of the names of associate editors and special contributors, and refers to "more than two hundred specialists in the various departments," who assisted in the collaboration of the work. In the partial printed list of associate editors are the names of two M. D.'s, one of them an eminent professor of clinical surgery in Philadelphia, now deceased.

After giving the definition of the word in Volume I of the American Dictionary and the descriptive article from which I have just quoted, the subject is again considered from the cyclopedic standpoint in Volume XI, and plainly a different associate editor or special contributor wrote the latter article, for truly he says, among other things: "Recurrent appendicitis is that form in which attacks of moderate severity occur at intervals, with intervening periods of comparative (or entire) freedom from recognizable intestinal lesion."

Of course, if, according to special contributor No. 1, "death is sure to follow promptly on neglect to remove the appendix" when attacked by inflammation, then contributor No. 2 is confronted, in his reference to *recurrent* appendicitis, with the dilemma of having to resurrect a dead man in order to find an example of a second or later attack in the same individual. The unreasonableness of the statement of the writer first quoted, that if removal of the appendix be neglected in an attack of appendicitis, "death is sure to follow promptly," is thus made apparent, else how could a second attack of appendicitis occur in an individual patient, as in our own observation we know it often does.

Writer No. 2, in discussing treatment, says: "Medical treatment, directed to the cleansing of the cœcum and large intestines, with local and general methods of allaying the inflammation, very frequently results in speedy cure if begun in time. This plan of relief is growing in favor as a result of experience, although some practitioners recommend excision of the appendix as the only positive cure, and also as a preventive."

The articles from which the foregoing extracts are taken, though unsigned, were plainly written by medical men selected as associate editors of a popular cyclopedia because of their acknowledged standing in the profession. My only excuse for quoting these conflicting and, indeed, irreconcilable statements, is that they faithfully reflect the chaotic and equally irreconcilable views as to the treatment of appendicitis which are to be found in text-books and special monographs of today, issued by every publisher of medical works, as well as in current discussions before medical associations. When doctors differ, who shall decide? My own veiws as to the best management of an acute attack of appendicitis, which I propose directly to lay before you, differ in some more or less important detail from those of every writer on the subject after whom I have read, but they do not differ in any essential detail from the treatment of idiopathic peritonitis as formulated and practiced by Sir Thomas Watson, Dr. Alonzo Clark, and Professor Austin Flint, Sr. So, without any claim whatever to having discovered anything new, I wish to call your attention to the proven merits of an old treatment for an old disease which for scarce ten years has been called by a new name.

John B. Murphy in his latest discussion of the subject (the November volume, 1902, of the American Medicine Series, Chicago), correctly states that the chief cause of mortality in appendicitis is from the complication of peritonitis. Occasionally a death occurs from septic phlebitis, followed by pyæmia or heart clot, rarely abscess formation and the consequent burrowing of pus, followed by chronic suppurative cachexia; but almost always, with very infrequent exceptions, a fatal termination is due to acute septic general peritonitis, or to plastic peritonitis with obstruction of the bowels.

It had long been known by the older teachers of internal medicine that general peritonitis was very commonly due to an extension of inflammatory action from a typhlitis, or perityphlitis, in the right iliac fossa. In fatal cases a post mortem had commonly shown a perforation of the cœcum and a fecal abscess alongside, from which the general peritonical cavity became involved later. And they formulated a systematized treatment for peritonitis calling for absolute physical rest in the recumbent posture, partial or complete abstinence from food, the scrupulous avoidance of all cathartics, and opium to the point of semi-narcosis, which, in all but the most advanced stages of general perforative peritonitis, had robbed the disease of its previous high mortality rate. Then came the discovery, scarce ten years ago, that the starting point of all this pathologic train was in the vermiform appendix, and not in the cœcum at all; that the primary involvement was an appendicitis, while typhlitis or perityphlitis was secondary. While this interesting discovery increased the sum of human knowledge, certain unfounded, though tolerably plausible, deductions were drawn therefrom, which caused the medical profession to lose sight of well-established principles and to very generally adopt a radical departure therefrom in the treatment of a circumscribed peritonitis, whose great danger was that it could so easily become diffused.

The first erroneous doctrine was as to the exciting cause of inflammation in the appendix. Except in those rare cases where a lodged grapeseed or a cherry stone was perhaps the efficient factor, it was attributed to undigested food, or an accumulation of fecal matter lodged in the cœcum, which, by producing a venous stasis, or by affording pabulum to various septic germs, originated the inflammatory process in the appendix. What could seem more plausible, then, than by brisk saline cathartics to soften down and dislodge the offending mass, thus removing the exciting cause of the trouble? And in cases of simple appendicular colic, or even of catarrhal appendicitis affecting at most the inner and middle coats of the organ, this treatment *is* followed usually by a cessation of symptoms. Professor Anders, of Philadelphia, in his text-book on the Practice of Medicine, warmly advocates saline cathartics at the beginning of treatment. But there are two considerations which should place an absolute ban on any kind of cathartics in this disease. The first is that typhilitis stercoralis, as it used to be called, or fecal impaction in the cœcum coincident with attacks of appendicitis, is an imaginary pathologic condition which many thousands of abdominal sections have shown does not exist. But the all-sufficient objection to the use of a cathartic in a clearly diagnosticated attack of appendicitis is that the vital danger from the attack lies in the transformation of a local into a general peritonitis; and every cathartic, by increasing peristalsis, tends to this disastrous result.

Another plausible notion became current on the general adoption of the new word appendicitis, and the abandonment, in America, at least, of the older term, perityphlitis. Let me here mention, in passing, that the thirteenth and latest edition of the German professor, Strumpell's, Practice, still names the disease perityphlitis, and the name appendicitis is inserted by Dr. Shattuck, the American editor, in brackets. The older name, with its more accurate description of the pathologic condition, in every well-defined attack, threatens, not only to go out of use, but to be forgotten, with all the pathology that it implies by the younger generation of American doctors just coming on the stage. Yet King Edward was operated on for *perityphlitis* by Sir Frederick Treves, who didn't even hunt for the appendix while draining the abscess, and the almost forgotten word had to be translated into appendicitis in the cablegram so that American readers might know what it was the king had.

But, going back and taking up the thread where we left off, the new word appendicitis, which indicates the starting point, had soon ccme in America to supercede the older term, perityphlitis, which better described the pathology of every well-developed attack; and what more natural than the proposal, and its immediate acceptance, *everywhere* and under *all* circumstances, to extirpate this useless, functionless, and, indeed, dangerous organ in the human economy? Nobody knows what it is there for. It is a rudimentary process, it is said, which was left over by the slipping of a cog at some stage of the evolution of man from the monkey. If general peritonitis, which is so fatal, be an extension from perityphlitis, which is tolerably serious, and the latter have its origin in the appendix, which nobody disputes, why not—to use the langage of one of my Knickerbocker patients, forcible but somewhat inelegant—"*Why not* cut the dern thing out"?

On this point let me say in all seriousness that our lack of knowledge as to the functions of an organ in no degree disproves that it may have a function, and a very important one, perhaps, indispensable, *it may be,* to the maintenance of good health, *if not of* life. When I first read Dalton's Physiology, the thyroid and suprarenal glands were also functionless, as were all the other ductless glands so far as anybody knew. But of these two it is now known that they secrete substances without whose constant presence the organism deteriorates in vitality and falls into sickness and decay. A sufficient time has not *yet* elapsed, it may plausibly be suggested, since the first surgical operation for removal of the appendix a few short years ago to justify the assertion in any single case that it *never* will be missed. And, while I readily admit that, in the light of our present knowledge, an appendix which is sufficiently diseased to occasion repeated attacks of recurrent appendicitis, with all the hazard to life which these imply, would better be removed; yet such a decision *may* be but a choice of the lesser of two evils. How many very robust people do you know from whom the appendix has been extirpated? On the other hand, do you not know a number who *are* robust, in spite of carrying about with them an appendix which has once upon a time given them trouble? I merely wish to throw out these suggestions as food for thought. You observe they are not in harmony with the views of some surgeons, who would ablate the normal organ while doing a laparotomy for some entirely different trouble lest it might sometime in the future become diseased.

There are surgeons not a few who strenuously deny that an attack of appendicitis may ever be treated medically with propriety. They teach that when the diagnosis of appendicitis is made operation should follow at once, whether it be the first day of the attack, the third day, the tenth day, or any other day. When Dr. Deaver, of Philadelphia, who operated 416 times last year (Report to the American Medical Association in 1902) and who has operated more than 4000 times all told—when Dr. Deaver promulgates this teaching oracularly and as one speaking by authority, those of us who are not exclusively surgeons, but simply general practitioners, could all surrender our divergent views and accept the teaching as being *ex cathedra* and practically infallible, were it not that Dr. Ochsner, of Chicago, comes along and proves by Dr. Deaver's statistics, as well as his own, that, after an acute attack has lasted thirty-six hours or more, operative measures are followed by a much higher rate of mortality than when the patient is treated medically until the acute stage of peritonitis, whether local or general, is tided over. And so the general practitioner breathes a sigh of relief, and realizes that he is not wholly *anathema maranatha* for having tided over many of these acute attacks by medical measures solely, instead of following the doctrine which, of late years and until Ochsner's writings, was hardly disputed, except perhaps in a timid whisper that he was virtually committing malpractice if he failed to operate whenever the diagnosis was made.

Ochsner is reporting a lower mortality rate from his operations than Deaver claims. The latter, as I said, operates at any stage of the attack; the former only in the first thirty-six hours or in the interval, after employing medical means to conduct the patient over the stage of local or general peritonitis if seen later than thirty-six hours after the beginning of the attack. Ochsner's treatment of the acute stage consists of:

1. *Entire abstinence* from all food by the mouth, both solids and liquids; because food in the stomach and small intestines causes necessarily more or less peristalsis, which tends to generalize a local peritonitis already existing and develop it when none exists.

2. Exclusive rectal alimentation every four hours by using small quantities of predigested liquid food, diluted with not exceeding four ounces of water.

3. Gastric lavage to remove the undigested food which may already exist in the stomach and small bowels, after which, he asserts, peristalsis and pain subside; and

4. Absolute rest in the recumbent posture.

He strongly discountenances in these cases the use of cathartics of all kinds, as they aggravate peristalsis and broaden the area of an existing peritonitis.

Under this plan a much larger percentage of cases of appendicitis recover than is claimed by any surgeon, Deaver included, who operates during the height of the attack; that is, after thirty-six hours have elapsed from the beginning.

In such of his writings on the subject as have met my eye, including his new work on clinical surgery, Ochsner has seemed to me strangely silent as to the use of opiates. For just as strenuously as Ochsner opposes all cathartics at all stages of the disease, with equal vehemence is the use of opiates opposed by Deaver and his following.

Until Ochsner, the surgeon, first advanced his new method, some eighteen months ago, of tiding over by medical means exclusively all cases seen during the acute stage after the first thirty-six hours of the attack, the medical treatment recommended by other authorities for an attack of appendicitis has fallen into one or the other of two categories:

The first method, in point of time, was the opiate treatment; and this was recommended by its advocates as a fundamental proposition without any very definite agreement as to other details. Many authors began with a cathartic in order to empty the cœcum, which was supposed to be clogged with fecal contents, reserving the administration of opiates until after preliminary brisk catharsis. Others discountenanced cathartics at any stage of the attack, and relied on opiates from the beginning. Osler, up to the fourth edition of his Practice, advocated opiates, and strongly opposed cathartics as tending to aggravate the existing or threatening peritonitis. Holt, in his Diseases of Children, first edition, taught the same doctrine: both of them advised clysters every day to move the bowels; Holt says give them twice a day. But in the fourth edition of Osler's Practice, published in July, 1902, the author turns these cases over to the surgeon, with the remark that "there is no medical treatment for appendicitis." Perhaps if Osler, the honored professor of internal medicine, had refrained from his daily administration of an enema, which excites the dreaded peristalsis in but a somewhat lesser degree than do the cathartics which he for this very reason so strongly condemns, he might even yet perhaps have continued to advocate a medical plan of treatment, at least until Ochsner, the surgeon, had come to his reinforcement. But, from the point of view of your essayist, all the benefits of the opiate treatment, which he advocated until recently on the ground that it checks peristalsis, could easily be canceled by a daily clyster, which can move the bowels only by exciting the same peristalsis he so much dreads. So as Osler, the physician, in his latest teachings, goes across the stage into the ranks of the operative surgeons, Ochsner, the surgeon, comes back into the camp of non-operating physicians, at the severest stage of the attack. At this stage of shifting sentiment and changing opinion you can almost hear the prompter say: "Swing corners! balance all! ladies change!" etc.

But while Osler says in his latest edition: "There is no medical treatment for appendicitis," he should not be misunderstood as saying there is no medical treatment for *an attack* of appendicitis. He still tells you how to treat peritonitis with opium; and peritonitis, according to Murphy, the surgeon, is the chief cause of mortality in these attacks. The uproar of the Philadelphia surgeons, denouncing opium in cases of appendicitis, denouncing everything, indeed, except the knife, had reached Baltimore (the two cities are only about 100 miles apart), and the din was so deafening that Osler got rattled. He'll come around all right in his next edition about two years hence.

The objection made to the use of opium by Dr. Deaver, who insists on operating whenever the patient is seen and the diagnosis is made, is that opium by soothing pain may obscure the diagnosis. And it may as well be admitted that, if the knife be demanded as the only proper method of treatment in any and every attack of appendicitis and at any and every stage of the disease, whether progressing unfavorably toward a general peritonitis or favorably toward a cessation of all inflammatory action, then opium is quite out of place in these attacks; for its judicious administration after the diagnosis has been established beyond a peradventure will in more than 90 per cent of cases encountered, if aided by suitable adjuvant measures, tide the sufferer over the danger period of an acute attack, until such time as an operation may remove the appendix in the interval between attacks with a mortality rate which, according to Ochsner, should not exceed one death in 500 operations.

It seems a remarkable thing to me that Dr. Deaver, who will not hear to the use of an opiate in an attack of appendicitis, does recognize that there *is* a medical treatment used in such cases, which consists in the administration of repeated doses of epsom salts. While he does not approve of this treatment, he nevertheless recognizes it as a treatment employed by medical practitioners. Indeed, in his latest paper, read last year before the American Medical

Association, he refers to the use of repeated cathartics as being "*the* medical treatment of appendicitis"; and says, doubtless with much truth, that it is followed by a higher mortality rate than are operative measures. A medical practitioner in Philadelphia who would give a dose of opium or morphine to a patient suffering from appendicitis would probably do it on the sly for fear of getting run out of town. At any rate, Dr. Deaver doesn't know of any other medical treatment of the disease—you would think from his latest paper he had never heard of any other—than by the use of saline cathartics, notwithstanding all the strictly medical authorities whom I have been able to find, with the single exception of Professor Anders, of Philadelphia, are unanimous in opposing cathartics and in favor of opium.

The saline cathartic treatment is justified on two grounds: It empties the cœcum of its fecal contents, which were formerly supposed, erroneously, however, to be the usual accompaniment and a frequent existing cause of inflammation of the vermiform process; and it was claimed to be efficient in combatting peritonitis because it was advocated for this purpose by Lawson Tait following his laparotomies. Saline cathartics served Tait well in his cases because, for one reason, he always left the alimentary canal impervious before closing the abdominal incision, while in perforative peritonitis, developing from appendicitis, the opposite condition prevails; and any cathartic, saline of otherwise, must in such cases aggravate an existing fecal infection of the peritoneal sack.

With Dr. Ochsner's medical treatment of an acute attack of appendicitis, I have had no experience in so far as gastric lavage is concerned. There is one class of cases in which I propose to try it when opportunity offers. Deaver calls attention to the danger in these cases of a plastic peritonitis which may bind down a coil of intestine, thus block its lumen, and produce a fatal obstruction of the bowels. Of course, he tries to remedy this otherwise fatal condition by operation, but the mortality he admits is high, and, worst of all, the condition tends to recur even after an otherwise successful operation. In a number of such instances reported in his last series of 416 cases, after the adhesions had been released, after the obstruction has been overcome, after the lumen of the bowel had been made patent, the plastic peritonitis persisted, or recurred, again causing obstruction; and the mortality following the second operation was 60 per cent. In the discussion following the reading of Deaver's paper, Ochsner led, and claimed that his treatment of the acute stage of appendicitis by gastric lavage, etc., would cause the opening up of an obstructed bowel. And this claim it is which causes me to resolve to try his treatment in the next suitable case. I fear Ochsner is too sanguine in his claims on this point, for he admits that he does fail to tide some acute cases over till a suitable time for the late operation, and that some do die. My own observation is that all fatal cases of appendicitis complicated by peritonitis which I have seen developed apparently complete obstruction before death.

Then Ochsner admits that his treatment by gastric lavage is not well adapted to children, who object to the use of the stomach tube so strenuously as to make its use generally unsuitable in childhood. In his Clinical Surgery, recently published, there seems to be a hiatus just here; for, after admitting that the general rules he has laid down arė inapplicable in the case of children, he leaves us to guess as to what alternative plan he adopts. It can not be that he operates in the acute stage with children, when he strongly discountenances operation at this period in adult patients. But, by his silence on this point, he leaves us guessing; and my guess is that he would not fail in these cases to give an opiate, and repeat it as often as might seem necessary, though he does not say so. But he does say in lecturing on a patient with fulminating appendicitis on whom he successfully operated ten hours after the attack began, that, after he had in consultation agreed to the diagnosis, and before starting the patient to the hospital for operation, a hypodermic of morphine has been administered, as the patient seemed on the point of convulsions from the agony of his suffering. He does not say who gave this hypodermic; he merely relates the fact, and sounds no note of disapproval nor warning against its repetition at a later stage of the attack when operation would be inadvisable.

The thanks of the medical profession, both physicians and surgeons, and of the laity as well, are due to Dr. Ochsner for showing that the mortality from operative measures during the acute stage, after the first thirty-six-hour period has passed, even at the hands of the most skilled and experienced surgeons, Dr. Deaver included, is several times as great as attends on his method, by medical means only, and without availing himself of the systematic use of opium in tiding them over until the acuteness of the attack has subsided, when he removes the appendix at a selected time with a mortality rate of less than one death from 500 operations.

And, though I have seen and treated less than a tithe of the number of cases recorded by this able and justly renowned surgeon, and though I have no carefully recorded tabulated statistical records going back over a period in general practice of nearly twentysix years,—going back to a time when appendicitis, though occurring just as frequently as nowadeys, was known by a different name, —I am sincerely of the opinion that the results I have observed were even better under the systematized method of treatment which I have of late years come to use than those even which Dr. Ochsner reports. I strongly suspect that the addition of Ochsner's plan of gastric lavage to the opium treatment as I have used it will, in those cases where undigested food lies in the stomach or small bowels, still farther reduce the mortality rate.

With Ochsner, I insist on: (1) Absolute rest in the recumbent posture. This appeals to good judgment as being rational; (2) The patient may take no food, either liquid or solid, by the mouth, during the entire acute attack. It can not be emphasized too strongly that a little food, even a thin soup, taken by the mouth, not only may, but probably will, by causing persistalsis, intensify all the dangers of the attack. Ochsner advises a half ounce of predigested liquid food, diluted with sufficient water to make four ounces, to be administered by enema every four hours. I have not used this method of feeding in these cases because it has not seemed necessary during the opium treatment as I employ it, since the patient's strength is very well conserved for a week's time by opium and rest. Ochsner admits that children object to the nutrient enemata about as much as they do to the stomach tube, thus making his method inapplicable to this large class of cases. The happiest results that I have seen in my practice have been in this class of cases—children —and without any feeding whatever, by rectum or otherwise, during the height of the attack. No patient will starve to death in eight days if stimulated the whole time by full doses of opium, and thereby held in a condition of complete physical rest. They do not for a week begin to feel the returning sensation of hunger. Until they do, I think they need no food; but cracked ice and very small quantities of water to allay the thirst, being absorbed from the stomach, will do no harm.

The radically new principle of Ochsner's treatment consists in washing out the contents of the stomach, to check vomiting in certain cases, and to arrest persistalsis in all. The method is rational and should be tested fully, but with every attention to other circumstantial details.

You all know the opium treatment of peritonitis. That is the treatment which I have found curative in those attacks which originate from a diseased appendix. But there are certain principles which must be observed to get the full benefit of the opiates. Opium in pill, powder, or tincture may be used, though of late years I use morphine with the same good results. It must be administered at least every two hours by the clock if the patient be awake, and preferably by the mouth, after the first acute suffering of the peritonitis has been soothed by hypodermics. The only discretion to be allowed the nurse is as to the size of the dose; the two-hourly interval *never* being exceeded if the patient be awake. It is not enough to direct the remedy be administered every two hours if the patient suffers pain. The patient *should never* suffer pain in a case of this kind after you have ascertained the quantity of morphine which will keep him dozing for the period of two hours. Pain indicates a recrudescense of the peritonitis. Pain is due to recommencing peristalsis, which *must* be held in check if you would prevent further extension of the inflammation. Tenderness of the abdomen on pressure persists for eight days in the most favorable progress of the case. A sense of soreness, even when lying quiet, is felt for five or six days, for the pulsation of arterial vessels and the involuntary incursions of the diaphragm in breathing can not be so well controlled as the peristaltic motion of the bowels by opiates in full doses. But, if the nurse be directed to give the remedy when needed to control pain, then the pain will needlessly recur when it should not, and will, when once started, continue for an hour or more before it is again allayed. Patients *die* of *pain* under these circumstances. Pain which was already agonizing, if it recur and be permitted to recur again, will so depress the vitality that the patient can not rally. If we wait until the patient gives evidence of pain before giving each dose of the remedy, we but tantalize the sufferer, and this plan of administering the remedy in peritonitis will inevitably bring the treatment into disrepute. Because it has so often been administered in this way is, in my judgment, the reason that it is by so many inadequately esteemed. Though I have from time to time modified the treatment in some minor detail, my consistent plan from the beginning has been to give the opiate every two hours if the patient be awake. If, when the dose is due, he is sleeping, wait till he awakes. Then, at once, give him the opiate, before the comfort caused by the previous dose has worn off. A condition, not only of continued freedom from pain is to be steadily maintained, but somnolence, a semi-narcosis, during the interval between doses, is to be the guide for regulating the quantity administered. The quantity of opium or morphine needed to maintain semi-narcosis varies with the individual and with the extent of peritoneum involved. A half grain of morphine every two hours is sufficient in many cases. Very much more than that I

have found necessary in some. The daily quantity taken by one patient of Dr. Alonzo Clark a half century ago sounds almost incredible, and a good recovery was had. Opium is a remedy which in all cases must be given for its effects. *The only safe guide, if the peritonitis be extensive, is to get and maintain semi-narcosis.* It will do no harm, and it *will* save life. Under this plan of treatment, tenderness in the right iliac region gradually subsides; and, by the end of eight days, I look for it to be gone. Not until pressure—gentle, steady, firm, deep pressure—over McBurney's point fails to elicit *tenderness* do I consider it safe to entirely discontinue the opiate, or to move the bowels by enema. A day or two earlier I sometimes permit the thinnest of soups, if hunger demands, but in the smallest quantities.

Under this plan of treatment I have met with no cases of fecal abscess. If such occurred, they found an outlet and drainage through the bowel and gave no trouble. The capacity of the peritoneum to absorb pus in certain quantities is, I am sure, not yet fully and duly appreciated. Dr. Deaver finds such frightful pathologic conditions in his cases, operated on after the second or third day, that he can not believe these inflammatory products can be removed by absorption, or by any other process than by incision and drainage. So he bluntly doubts that Ochsner is seeing as bad cases in Chicago as come to him in Philadelphia.

Medical and surgical sentiment as to the best management of acute appendicitis in not yet crystallized, nor will it be for a long time if one surgeon's *ipse dixit* may outweigh the careful observations of other surgeons of equal ability. But the fact seems clear, and statistics appear to prove it, that there is a time to operate and a time to abstain. That, while surgery offers the only cure for appendicitis, the judicious medical management of an acute attack will save life at a time when nothing else will. The physician and surgeon should co-operate in these cases, and the broadest spirit of tolerance is equally becoming in both.

